# Diabetes in the African-Caribbean and the Romford Experience (DARE): A Retrospective Study

**DOI:** 10.7759/cureus.100183

**Published:** 2025-12-27

**Authors:** Noha Meneissy, Adnan Abdullah, Bhavini Bhatt, Ehsan Shakoor, Furhana Hussein, Zahid Khan, Lina Eltayieb, Bashir Mahamud, Azhar Faisymohammed, Gideon Mlawa

**Affiliations:** 1 Endocrinology and Diabetes, Barking, Havering and Redbridge University Hospitals NHS Trust, London, GBR; 2 Acute Medicine, Barking, Havering and Redbridge University Hospitals NHS Trust, London, GBR; 3 Medicine, University College London Medical School, London, GBR; 4 Internal Medicine, Queen's Hospital, London, GBR; 5 Internal Medicine and Diabetes and Endocrinology, Barking, Havering and Redbridge University Hospitals NHS Trust, London, GBR; 6 Cardiology, Barts Health NHS Trust, London, GBR; 7 Medicine, Barking, Havering and Redbridge University Hospitals NHS Trust, London, GBR; 8 Internal Medicine, Barking, Havering and Redbridge University Hospitals NHS Trust, London, GBR

**Keywords:** afro-caribbeans, diabetes, diabetic ketoacidosis (dka), endocrine disorders, types 2 diabetes

## Abstract

Background: Diabetes mellitus is a significant public health challenge in the United Kingdom, with higher prevalence and complication rates among minority ethnic groups, especially those of African and Caribbean descent. Evidence shows that these populations have an increased risk of developing type 2 diabetes and related metabolic issues, often presenting late with acute hyperglycemic emergencies like diabetic ketoacidosis (DKA) or hyperosmolar hyperglycemic state (HHS). Understanding patterns of disease onset and early management in these communities is crucial for reducing health disparities and improving outcomes.

Aim: The DARE study aimed to describe the clinical characteristics, presentation patterns, diabetes subtypes, and early management of Afro-Caribbean patients admitted to a hospital in Romford with diabetes or diabetes-related complications. The objectives were to identify the prevalence of diabetes subtypes, assess demographic trends, document acute presentations, and evaluate early inpatient management.

Methods: This retrospective observational study focused on Afro-Caribbean adults who were admitted to Queen’s Hospital, Romford, for diabetes or related complications from January 2020 to December 2024. Eligible patients were identified through CareFlow Connect, the London Care Record, and EPRO, applying specific inclusion criteria. In total, 52 individuals comprised the final study group. Descriptive statistics were used to summarise the data, with analysis performed in Microsoft Excel (Redmond, USA) and Google Sheets (California, USA).

Results: The study cohort included 52 patients (mean age 46 years, range 18 to 86), of whom 33 (63.5%) were male. Type 2 diabetes was the most prevalent subtype (39 of 52, 75.0%), followed by type 1 diabetes (7 of 52, 13.5%) and latent autoimmune diabetes in adults (LADA) (5 of 52, 9.6%). Acute metabolic emergencies were common: diabetic ketoacidosis (DKA) occurred in 20 of 52 patients (38.5%), hyperosmolar hyperglycaemic state (HHS) in 13 of 52 (25.0%), and a mixed DKA/HHS presentation in 1 of 52 (1.9%). Among patients younger than 30 years, 4 of 9 (44.4%) presented with DKA. Non-emergency presentations included hyperglycaemia without ketosis (n = 9) and polyuria or polydipsia (n = 4). Insulin use was frequently documented, with both long-acting and short-acting insulin each recorded in 28 of 52 patients (53.8%). Six patients (11.5%) were identified as deceased in available hospital-linked records; information regarding place of death and complete follow-up outside these records was unavailable.

Conclusion: In this admission-based cohort of Afro-Caribbean adults in Romford, acute hyperglycaemic emergencies (DKA/HHS) were common at first hospital presentation, and insulin therapy was frequently recorded. These findings are descriptive and hypothesis-generating and support the need for locally responsive, culturally informed approaches to earlier identification and continuity of diabetes care in high-risk communities. Further multi-centre studies with standardised definitions and improved linkage to community and primary care records are warranted to clarify pathways to diagnosis, barriers to timely care, and the impact and cost-effectiveness of targeted interventions.

## Introduction

Diabetes mellitus is a long-term health condition marked by raised blood sugar levels. This happens either because not enough insulin is produced or because the body cannot use insulin properly, or sometimes a combination of both [1]. In the United Kingdom, diabetes now affects more people each year, but not everyone is impacted in the same way. People from minority ethnic backgrounds, especially those of Black and South Asian descent, are more likely to develop diabetes than the White British population [2]. Studies show that type 2 diabetes is almost twice as common in these groups compared to their White peers [3].

One worrying aspect is that people from minority ethnic communities are sometimes first diagnosed with diabetes when they develop a serious complication, such as diabetic ketoacidosis (DKA) or a hyperosmolar hyperglycaemic state (HHS). This often means there were delays in diagnosis or challenges in accessing healthcare [4]. African-Caribbean people are particularly at risk, with factors like obesity, family history, low vitamin D, insulin resistance, and social disadvantage all playing a part [5,6]. Complications from diabetes, such as problems with the eyes, kidneys, or nerves, also seem to occur more often in these groups than among White patients [6,7].

Looking at the bigger picture, studies following people over many years have highlighted just how much more common diabetes is in some groups. For example, the Southall and Brent REvisited (SABRE) study found that by the age of 80, nearly half of South Asian, African, and African-Caribbean people had developed type 2 diabetes, compared with only about one in five people of European heritage [8]. This shows that the risk is much higher in some communities and highlights why we need prevention and screening tailored to different backgrounds, with fair access for all. This is the reason behind the DARE study, which set out to examine how common diabetes is among African-Caribbean residents in Romford, how people present, and the complications they face. Romford is a diverse area, according to the 2021 Census, around 8% of the local population identify as Black, Black British, Black Welsh, Caribbean, or African [9]. Knowing more about this community helps determine which services and support are most needed.

Aim

To provide a descriptive, hypothesis-generating characterisation of diabetes subtype, patterns of presentation (including DKA/HHS), and recorded glucose-lowering therapy among Afro-Caribbean adults admitted with diabetes or diabetes-related complications at Queen’s Hospital, Romford (January 2020-December 2024).

Objectives

To describe baseline characteristics at admission, including age and sex; to classify diabetes subtype as recorded in clinical documentation (type 1, type 2, LADA, or other specified forms) and report the proportion of each subtype; to quantify patterns of presentation, including DKA, HHS, mixed DKA/HHS, and non-emergency presentations, and describe these by age group (for example, ≤30 years versus >30 years); and to describe the glucose-lowering therapy documented in the clinical record at the time of admission and/or discharge, including insulin regimens and non-insulin agents.

## Materials and methods

This retrospective observational study was conducted at Queen’s Hospital, Romford, and focused on Afro-Caribbean adults admitted with diabetes mellitus or diabetes-related complications between January 2020 and December 2024. The study was designed as a descriptive, hypothesis-generating evaluation to characterise patterns of clinical presentation, including acute metabolic emergencies, the distribution of diabetes subtypes, and the use of glucose-lowering therapies within this ethnic group.

Case identification and data sources

**Figure 1:**
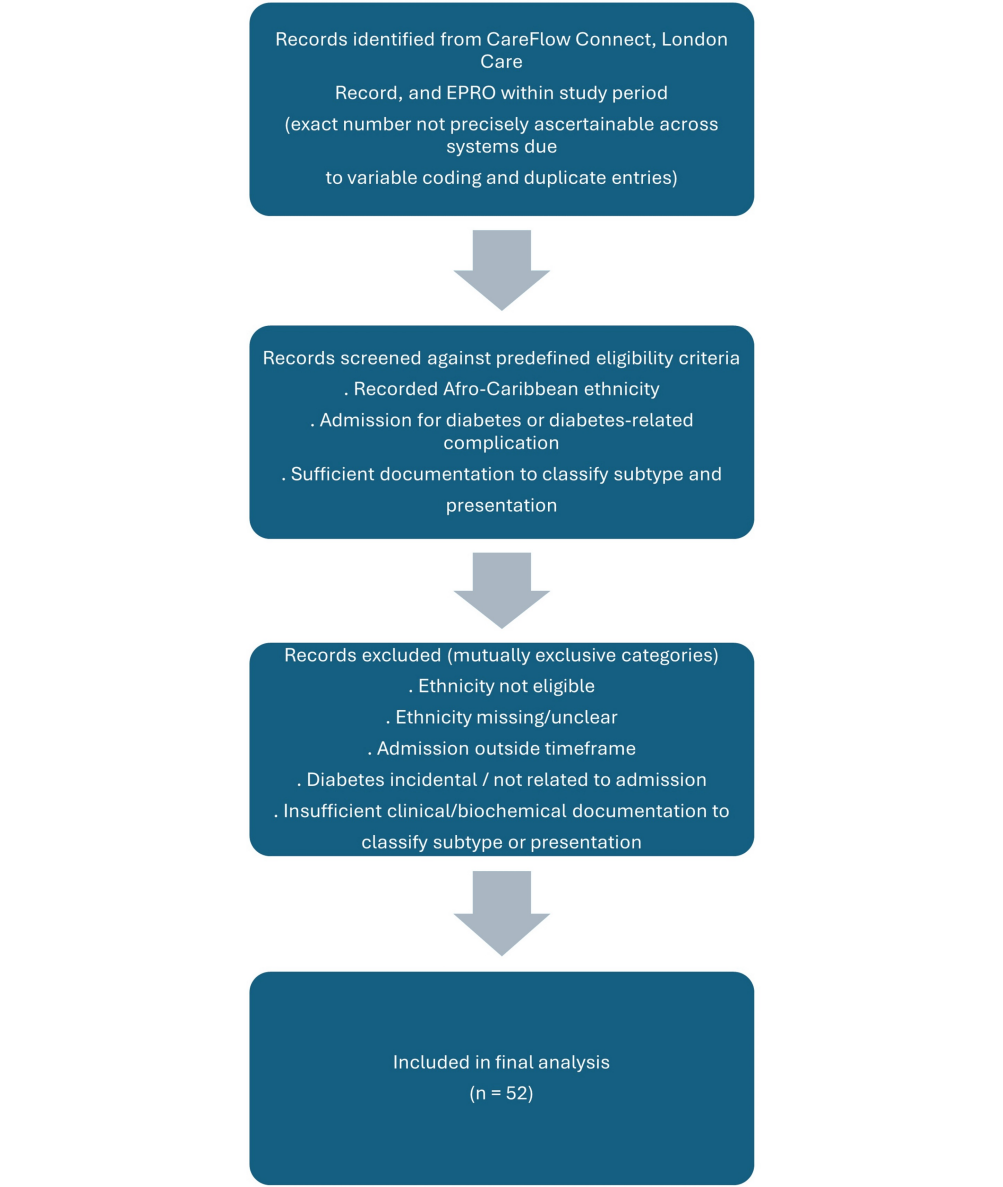
Flow diagram summarising identification, screening, predefined exclusion categories, and inclusion of the final cohort (n=52). Exact denominators for all diabetes-related admissions and exclusions could not be enumerated reliably across the three electronic systems due to variable coding and duplicate entries.

All adult admissions during the study period with documented diabetes or diabetes-related complications were screened for inclusion. Potential cases were identified using three electronic systems: CareFlow Connect (admission records), the London Care Record (regional summaries and diabetes diagnoses), and the EPRO database (discharge documentation and clinical interventions). Each system contributed specific data: CareFlow Connect provided admission data, the London Care Record provided regional records and coded ethnicity, and the EPRO database provided discharge and episode-of-care details. Exact denominators for all diabetes-related admissions could not be reliably determined across the three systems due to variable coding and duplicate entries. Therefore, the final included cohort (n=52) is reported, with exclusions described by predefined categories. A schematic flow diagram summarising the identification process and exclusion categories is presented in Figure 1.

Ethnicity was obtained from the electronic health record, as reported by patients at registration. No independent verification of ethnicity was performed.

Inclusion criteria

Patients were included if all of the following criteria were met: 1) recorded Afro-Caribbean ethnicity, 2) a documented diagnosis of diabetes mellitus (type 1, type 2, or other specified forms as recorded in the clinical record), and 3) admission to Queen’s Hospital within the study timeframe for diabetes or a diabetes-related complication.

Exclusion criteria

Records were excluded according to the following predefined categories: 1) ethnicity not eligible (not Afro-Caribbean), 2) ethnicity missing or unclear in available records, 3) admission outside the study timeframe, 4) diabetes documented as incidental to the admission (diabetes listed in past medical history but not relevant to the admission, and insufficient documentation for analysis), or insufficient medical information to determine the type of diabetes or to describe the initial presentation to the hospital (for example, inadequate details to identify diabetic ketoacidosis, hyperosmolar hyperglycemic state, or diabetes type).

Data collection

Data were extracted from electronic patient records and discharge documentation using a predefined data-extraction framework. Variables collected included demographics (age and sex), diabetes subtype (as recorded), presenting features, acute metabolic emergencies (diabetic ketoacidosis, hyperosmolar hyperglycaemic state, or mixed DKA/HHS, as documented), comorbidities when available, and recorded glucose-lowering therapy during the admission episode (insulin regimens and non-insulin agents).

Data governance

All extracted data were anonymised and stored securely in accordance with institutional information governance requirements. The study used routinely collected clinical data and involved no direct patient contact. Management was conducted under local arrangements for retrospective service evaluation.

Data analysis

We used Microsoft Excel and Google Sheets to review the data. Patient demographics, diabetes subtypes, primary health problems, comorbidities, and treatments were described using numbers and percentages for each category, with numerical details summarised accordingly.

## Results

**Figure 2:**
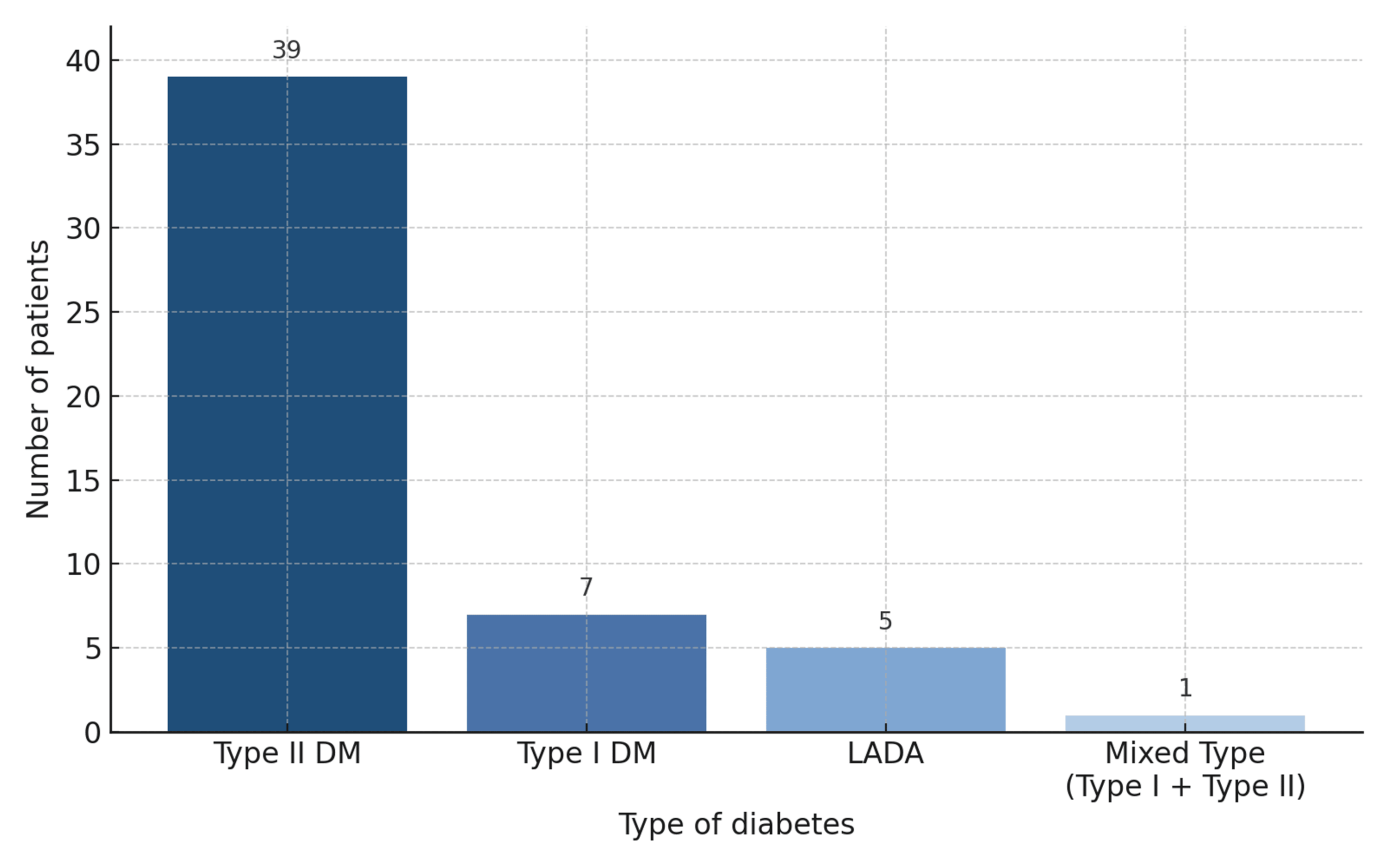
Distribution of diabetes subtypes in the Afro-Caribbean cohort.

The cohort comprised 52 patients aged 18-86 years (mean 46 years); 33 (63.5%) were male, and 19 (36.5%) were female. Type 2 diabetes mellitus was the most prevalent diagnosis, identified in 39 patients (75%). Type 1 diabetes was observed in seven patients (13.5%), while latent autoimmune diabetes in adults (LADA) was diagnosed in five patients (9.6%). Additionally, a mixed phenotype was recorded in one patient (1.9%). The distribution of diabetes subtypes is shown in Figure 2.

**Figure 3:**
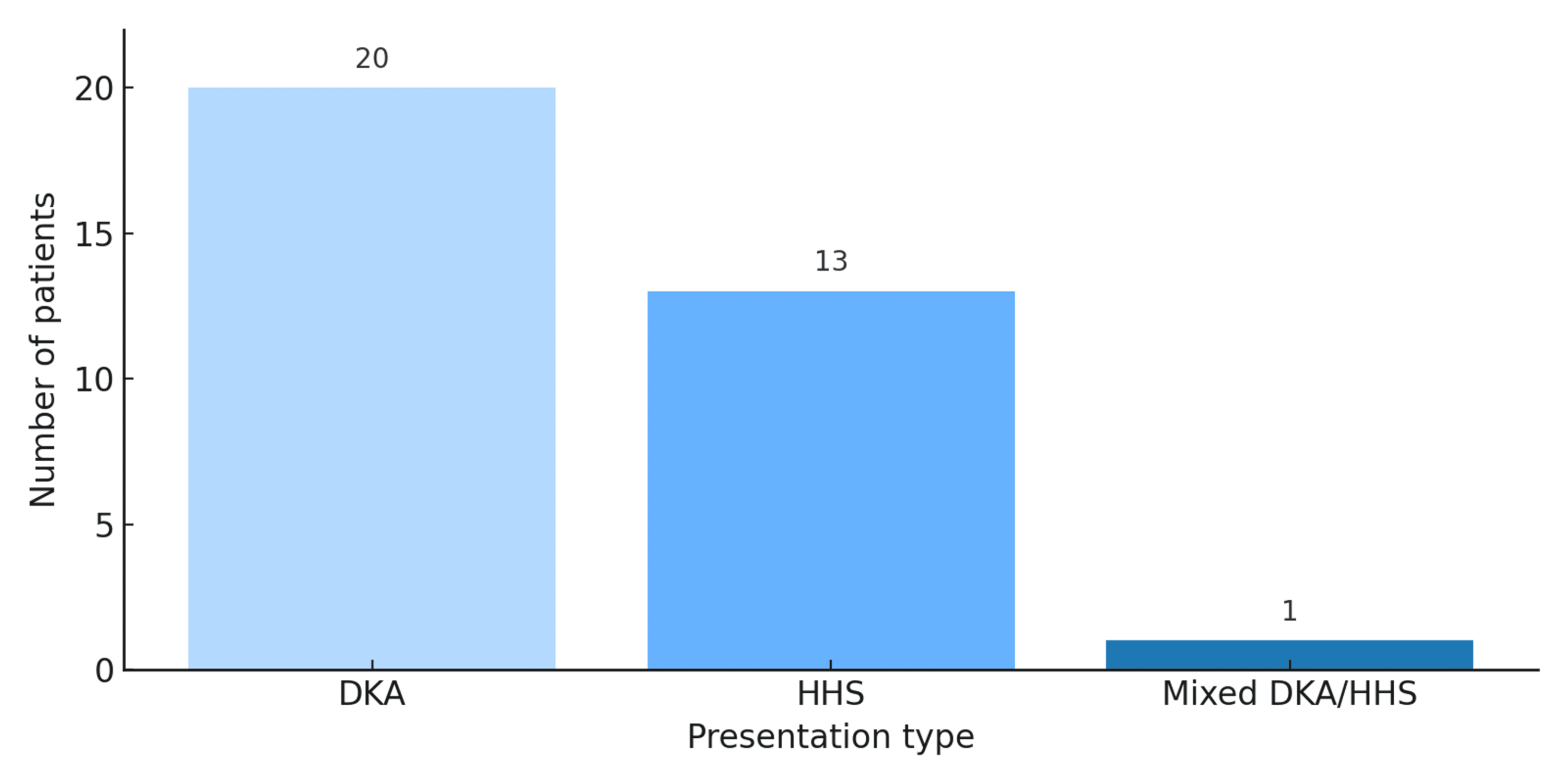
Presentation of acute metabolic emergencies.

The distribution of acute metabolic emergencies among Afro-Caribbean patients admitted with diabetes or diabetes-related complications showed that diabetic ketoacidosis (DKA) accounted for the highest proportion of acute presentations (n = 20), followed by hyperosmolar hyperglycaemic state (HHS) (n = 13). A small number of patients presented with a mixed DKA/HHS picture (n = 1). These findings demonstrate that acute metabolic decompensation accounts for a significant proportion of initial presentations within this cohort and is depicted in Figure 3.

In age-stratified descriptive analysis, among patients younger than 30 years, nearly half (4 of 9; 44%) presented with DKA, whereas the remaining five patients (56%) exhibited non-DKA presentations. These findings highlight an increased propensity for acute metabolic decompensation at initial presentation among younger Afro-Caribbean patients.

**Figure 4:**
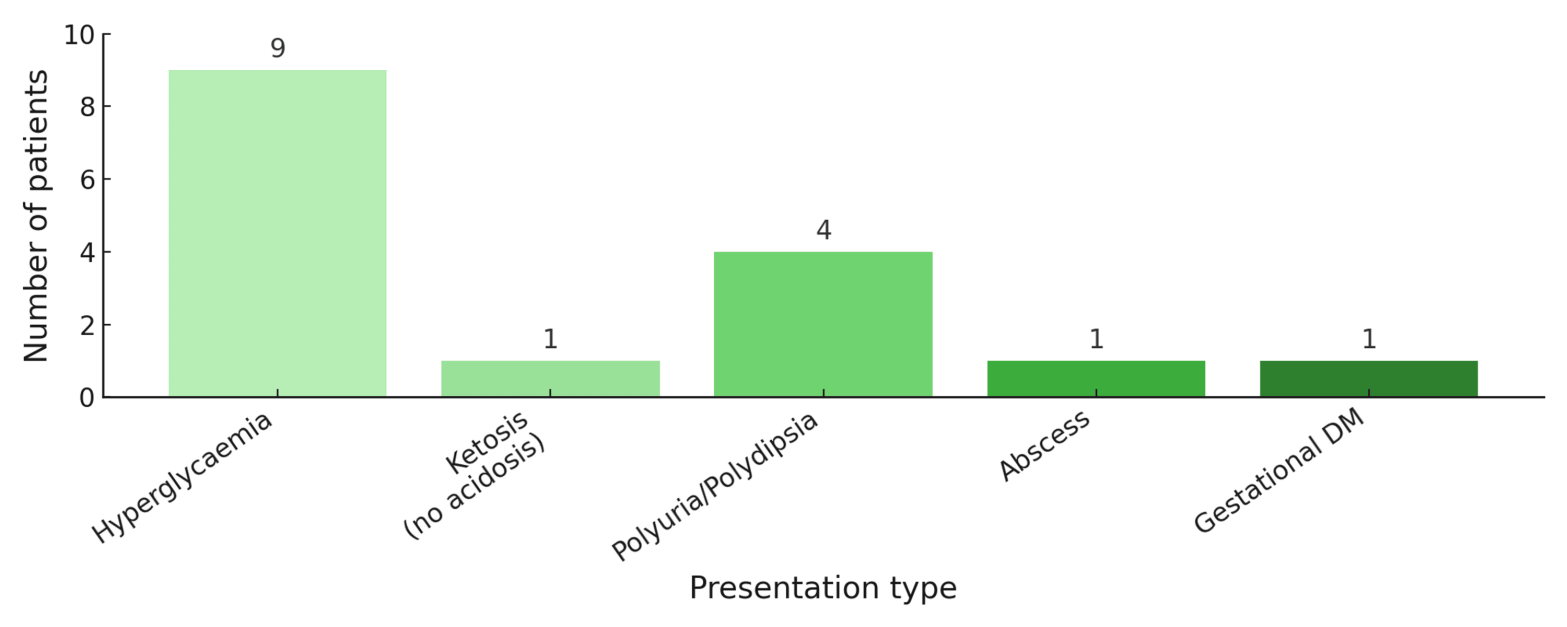
Summarising the non-acute presentation.

Non-emergency presentations included hyperglycaemia without ketosis (n=9), polyuria/polydipsia (n=4), ketosis without acidosis (n=1), soft-tissue abscess (n=1), and gestational diabetes (n=1) (Figure 4). Two presentations were not classifiable from the available documentation. 

Insulin was the most commonly recorded glucose-lowering therapy. Long-acting and short-acting insulin were each documented in 28/52 (53.8%), and mixed insulin in 1/52 (1.9%). Among non-insulin therapies, metformin was documented in 26/52 (50.0%), sulphonylureas in 8/52 (15.4%), Sodium/glucose cotransporter 2 (SGLT2) inhibitors in 5/52 (9.6%), gliptins in 5/52 (9.6%), and a GLP-1 receptor agonist in 1/52 (1.9%). Two patients (3.8%) had no glucose-lowering medication documented (Table 1). Several patients were on combination therapy.

**Table 1:** Recorded glucose-lowering therapy (n=52). SGLT2: Sodium/glucose cotransporter 2, GLP-1: Glucagon-like peptide 1 Patients may be on more than one therapy; percentages do not sum to 100%.

Medication Class	Number of Patients	Percentage (%)
Long-acting insulin	28	53.8
Short-acting insulin	28	53.8
Mixed insulin	1	1.9
Metformin	26	50.0
SGLT2 inhibitors	5	9.6
Sulphonylureas	8	15.4
GLP-1 receptor agonist	1	1.9
Gliptins	5	9.6
No medication	2	3.8

Follow-up outcomes

Follow-up information was obtained by reviewing available hospital-linked electronic records. Six patients (11.5%) were identified as deceased during the study period based on these records. However, the location of death (in-hospital or out-of-hospital) and comprehensive follow-up for patients outside hospital-linked systems could not be confirmed, limiting the completeness of these data. Therefore, mortality should be interpreted as deaths documented in the accessible records, recognising that this approach may not capture all deaths within the cohort.

When documentation permitted, diabetes and its complications were recorded as potential contributing factors to mortality. One death was associated with diabetic ketoacidosis (DKA). Other deaths were linked to diabetes-related complications, such as severe infection (for example, diabetic foot infection or sepsis), impaired wound healing, and cardiovascular events in individuals with diabetes.

During the same period, additional diabetes-related complications were documented for some patients in hospital-linked records, including lower-limb amputation, soft-tissue infection or abscess (including pilonidal abscess), and cardiovascular disease. These complications were identified through routine clinical care rather than a standardised or comprehensive prospective follow-up protocol. Therefore, unrecorded complications outside these records cannot be excluded.

## Discussion

Comparisons with national datasets indicate that the proportion of acute presentations observed in this cohort may exceed national expectations. The national data report diabetic ketoacidosis (DKA) admissions in England as incidence rates per person-years by diabetes type [10], whereas the present data reflect proportions within an admission-based, single-centre cohort. Therefore, these comparisons are context-specific, and differences in study design, populations, and denominators constrain direct comparability.

The frequent acute presentations in this cohort likely reflect missed opportunities for earlier diagnosis, delays in seeking care, or barriers to timely diabetes management. These factors were not directly measured in this dataset. UK studies have demonstrated ethnic disparities in diabetes monitoring and treatment, including lower use of specific agents and differences in annual reviews among Black individuals with type 2 diabetes [11]. These findings highlight the need to examine local diagnostic pathways and underlying reasons for acute presentation in Afro-Caribbean communities.

Type 2 diabetes was the predominant subtype in this cohort (75%), consistent with national patterns [11]. Latent autoimmune diabetes in adults (LADA) accounted for 9.6%, which is higher than typically observed in routine clinical practice. This finding suggests that less-recognised or atypical phenotypes may be present within minority populations. Subtype classification in this retrospective study relied on existing documentation and available tests, which may have resulted in misclassification [12]. Future studies employing standardised case definitions, including antibody and C-peptide data, are needed to clarify the prevalence of autoimmune or mixed phenotypes in similar cohorts.

Therapy patterns recorded in this cohort indicated a high reliance on insulin, with both long-acting and short-acting insulin documented in 53.8% of patients. This likely reflects the severity of presentations necessitating hospital admission and the frequency of acute metabolic emergencies, rather than long-term treatment requirements. When comparing these findings with UK prescribing patterns, it is essential to note that this cohort consists exclusively of admitted patients and does not represent community-managed diabetes populations. Nonetheless, these results underscore the importance of early identification and proactive escalation of care for high-risk individuals, supported by robust transitional planning between inpatient and community services.

Six patients (11.5%) were identified as deceased within hospital-linked records during the study period. However, information regarding the place of death and complete follow-up outside hospital-linked systems was unavailable. Mortality should therefore be interpreted as recorded deaths within accessible records rather than as a comprehensive estimate.

Strengths and limitations

This study examines an under-researched ethnic group in a specific UK locality, utilising hospital data to detail clinically relevant presentation patterns and treatments. It provides a structured account of acute metabolic emergencies and the distribution of diabetes subtypes in a cohort central to local service delivery.

Several limitations should be considered. The small sample size and retrospective, single-centre design limit generalisability and preclude causal inference. Ethnicity was derived from routinely recorded electronic health data, typically self-reported at registration, and was not independently validated, potentially leading to misclassification. Selection bias is possible, as inclusion required sufficient documentation to classify subtype and presentation; excluded records may have differed systematically in severity or presentation. Additionally, no internal comparator cohort from the same hospital was included. Comparisons in this study are therefore made between the study cohort and national data, which serve only as contextual references and are not directly comparable due to differences in underlying populations and denominators. Mortality and complications were ascertained from hospital-linked records; place of death and outcomes occurring solely outside these systems could not be confirmed, potentially underestimating event rates.

Recommendation

These findings demonstrate the need for targeted, culturally informed interventions to promote earlier diagnosis and prevent acute metabolic decompensation in Afro-Caribbean communities. Services should strengthen proactive case-finding in primary care for high-risk groups, provide culturally tailored education on hyperglycaemic symptoms and appropriate care-seeking, and improve coordination between acute services and community diabetes teams to reduce recurrent emergencies following discharge. Local prescribing audits can assess equitable access to evidence-based therapies, such as sodium-glucose cotransporter-2 (SGLT2) inhibitors and glucagon-like peptide-1 (GLP-1) receptor agonists, when clinically appropriate.

Future research directions

Future research should expand this evaluation through multicentre studies employing standardised definitions of diabetes subtypes and hyperglycaemic emergencies to determine whether similar admission patterns are observed across the UK. Qualitative research exploring patient experiences and barriers to timely diagnosis and care could elucidate pathways leading to acute presentation. Where feasible, linkage to primary care data would enable more comprehensive follow-up and improved ascertainment of outcomes, including mortality and complications. Further studies should assess the cost-effectiveness of earlier targeted screening and culturally adapted interventions to inform NHS service planning.

## Conclusions

The DARE study gives a descriptive overview of diabetes presentations and therapies recorded among Afro-Caribbean adults admitted to Romford with diabetes or related complications. In this cohort, many arrived with acute hyperglycaemic crises such as diabetic ketoacidosis (DKA) and hyperosmolar hyperglycaemic state (HHS). Insulin therapy was common at admission and discharge. These findings demonstrate the clinical severity observed in hospital care and underscore the need for earlier recognition and targeted prevention in high-risk communities. National datasets provide helpful context. However, differences in population and denominator sizes limit the validity of direct comparisons. This study lacked an internal comparator cohort. We did not measure factors such as diagnostic delays, barriers to care, or biological influences. The existing literature supports these as possible contributors, but the present data do not allow for conclusions.

Future research should involve larger, multi-centre studies with standardised definitions and enhanced linkage to community and primary care records to more accurately characterise diagnostic pathways, treatment trajectories, and outcomes. In the interim, key recommendations include delivering culturally informed education to patients and communities, strengthening primary care efforts to identify and manage diabetes early, and improving integration and communication between hospital and community diabetes services. These measures may reduce the risk of acute metabolic decompensation and address disparities in diabetes care.
